# The Cerebellum’s Orchestra: Understanding the Functional Connectivity of Its Lobes and Deep Nuclei in Coordination and Integration of Brain Networks

**DOI:** 10.3390/tomography9020072

**Published:** 2023-04-21

**Authors:** Adnan A. S. Alahmadi

**Affiliations:** Department of Radiologic Sciences, Faculty of Applied Medical Sciences, King Abdulaziz University, Jeddah, Saudi Arabia; aaalahmadi@kau.edu.sa

**Keywords:** cerebellum, functional connectivity, resting state fMRI, deep cerebellar nuclei

## Abstract

The cerebellum, a crucial brain region, significantly contributes to various brain functions. Although it occupies a small portion of the brain, it houses nearly half of the neurons in the nervous system. Previously thought to be solely involved in motor activities, the cerebellum has since been found to play a role in cognitive, sensory, and associative functions. To further elucidate the intricate neurophysiological characteristics of the cerebellum, we investigated the functional connectivity of cerebellar lobules and deep nuclei with 8 major functional brain networks in 198 healthy subjects. Our findings revealed both similarities and differences in the functional connectivity of key cerebellar lobules and nuclei. Despite robust functional connectivity among these lobules, our results demonstrated that they exhibit heterogeneous functional integration with different functional networks. For instance, lobules 4, 5, 6, and 8 were linked to sensorimotor networks, while lobules 1, 2, and 7 were associated with higher-order, non-motor, and complex functional networks. Notably, our study uncovered a lack of functional connectivity in lobule 3, strong connections between lobules 4 and 5 with the default mode networks, and connections between lobules 6 and 8 with the salience, dorsal attention, and visual networks. Additionally, we found that cerebellar nuclei, particularly the dentate cerebellar nuclei, were connected to sensorimotor, salience, language, and default-mode networks. This study provides valuable insights into the diverse functional roles of the cerebellum in cognitive processing.

## 1. Introduction

The cerebellum is one of the most important brain regions (lobules); it is sometimes considered another brain (in Latin, its name means ‘little brain’). The cerebellum was formerly believed to be exclusively involved in motor processes; however, research later showed that it was also involved in cognitive, sensory, and associative functions [1,2,3,4,5]. Most of our physiological functional understanding of the cerebellum is based on investigations using functional magnetic resonance imaging (fMRI) with a specific experimental task [6,7]. Such investigations are restricted by either the narrow focus of the study on the function of a single sub-cerebellar region, which limits our understanding of how other cerebellar regions behave physiologically, or their experimental design is limited to a single purpose and set of hypotheses.

Resting-state fMRI (rsfMRI), which assists in identifying functional integrations of brain regions while subjects are at rest (i.e., not performing experimental tasks), is one of the most sophisticated and effective techniques applied to improve our understanding of the complex organization and neurophysiological processes of the brain [8]. Because rsfMRI creates functional, integrative networks of various regions specific to brain functions, its results are more realistic in terms of the nuanced characterization of brain neurophysiology, making this method powerful [9]. Furthermore, this strategy is effective because it does not require individuals to complete a specific task, which makes it a desirable approach for patients who have complex neurodegenerative symptoms.

It is important to note that the cerebellum’s role in the overall functionality of the brain has been historically underestimated due to its relatively small size compared to other regions and traditional research methodologies. However, with the advent of advanced neuroimaging techniques such as rsfMRI, the scientific community has been presented with an opportunity to delve deeper into the cerebellum’s intricate connections and its vital contributions to numerous brain functions [8,9]. This increased focus on the cerebellum has led to a broader understanding of its significance in both normal and pathological conditions, which, in turn, has opened new avenues for therapeutic interventions.

Few previous studies have investigated cerebellar regions and their functional integrations, while very rare studies have looked at the physiological changes of the cerebellar nuclei, e.g., [2,10,11,12,13,14,15,16,17]. However, these studies either focused on single cerebellar regions or did not investigate the connectivity of the cerebellar lobules with functionally related regional networks (i.e., several brain regions that form a network responsible for a specific function). Therefore, the aim of this study was to investigate, in one single study using a large cohort, the functional connectivity of cerebellar lobules as well as the deep cerebellar nuclei (DCN), namely lobules 1–10 and 4 cerebellar nuclei: Interposed (emboliform and globose), fastigial, ventral dentate, and dorsal dentate nuclei. In addition, the integrative networks of various brain functions, such as default-mode, visual, sensorimotor, salience, dorsal attention, frontoparietal, cerebellar, and language networks, were selected as target networks of interest. The specific aim is to further improve the understanding of the complex neurophysiological formations of the cerebellum and its connections with other brain regions.

## 2. Methods

### 2.1. Data Source

For this investigation, 198 healthy volunteers were enlisted (171 were right-handed; range of age was 18–30; 123 were females). The Cambridge-Buckner dataset, a component of the 1000 Functional Connectomes Project (an open-access platform without any restrictions; see the IRP statement http://fcon_1000.projects.nitrc.org (accessed on 9 April 2022)), was used to collect and download the data [9].

### 2.2. Scanning Parameters

Each subject was diagnosed using a Siemens 3 Tesla Trim Trio scanner with the subsequent factors: rsfMRI using a T2* weighted Echo Planner Imaging (EPI) sequence with repetition time (TR) = 3000 ms; TE = 30 ms; number of slices = 47 interleaved axial slices to cover the whole brain including the cerebellum; voxel size: 3.0 × 3.0 × 3.0 mm^3^; number of time points (i.e., volumes) = 119 volumes; T1-weighted MPRAGE images with the following parameters: number of slices: 192; matrix size 144 × 192; voxel size: 1.20 × 1.00 × 1.33 mm^3^. Please see The Cambridge-Buckner dataset, which is a component of the 1000 Functional Connectomes Project (an open-access platform without any restrictions; see the IRP statement http://fcon_1000.projects.nitrc.org (accessed on 9 April 2022)); for more information, see [9].

### 2.3. Pre-Processing

MATLAB (R2020a) (MathWorks, Middlesex, Massachusetts, United States), CONN (https://web.conn-toolbox.org (accessed on 9 April 2022)) [18], and Statistical Parametric Mapping software (SPM12) (Univeristy College London, London, United Kingdom) [19,20,21,22] were used to analyze the whole functional integrations of the data. The rsfMRI images were pre-processed using customary methods. The pre-processing steps included slice timing adjustments to correct temporal differences in image acquisition between slices, functional volume realignments to account for head motion, normalization to a common space template using each subject’s structural data, outlier detection using CONN’s implanted artifact detection tools (ART), and smoothing the functional volumes with an 8 × 8 × 8 mm^3^ kernel. Additionally, blood-oxygen-level-dependent (BOLD) signal of rsfMRI was subjected to temporal processing employing data denoising to reduce the impact of artifacts and confounds. This included regressing out signals from white matter, cerebrospinal fluid (CSF), motion parameters, and scrubbing factors.

### 2.4. Selection of Regions of Interest

In this study, 10 cerebellar lobules in both hemispheres were identified as seeds. To accurately pinpoint these lobules, the Harvard–Oxford cortical and subcortical structural atlas was employed, which utilizes the statistical likelihood of numerous areas to precisely define each brain region. Additionally, four distinct cerebellar nuclei were determined through cytoarchitecture analysis and classified as Interposed (emboliform and globose), fastigial, ventral dentate, and dorsal dentate nuclei [23,24].

Furthermore, the target brain regions encompassed large groups of functional networks, as delineated by CONN. These networks were derived from the human connectome research project, which included 497 healthy participants (ICA). Among the functionally targeted networks were the default mode, attention, sensorimotor, visual, salience, dorsal attention, frontoparietal, cerebellar, and language networks. This comprehensive approach allowed for a thorough examination of the interplay between cerebellar lobules and nuclei with various functional networks, offering valuable insights into the cerebellum’s intricate involvement in diverse brain processes.

### 2.5. Statistical Analysis

The statistical analyses were performed at two statistical levels: subject and group levels. At the first level of analysis (subject’s level), matrices of regions of interest (ROI)-to-ROI connections between and among the termed ROIs were calculated for each participant using weighted general linear bivariate correlation models. Bivariate Fisher-transformed correlation coefficients between the pair ROI time series were used to build these matrices. The Fisher transformation, given by the equation z = 0.5 × ln((1 + r)/(1 − r)), where r is the correlation coefficient, allows for more accurate confidence intervals and hypothesis testing. The transformation converts Pearson’s correlation coefficients into z-scores, which follow a normal distribution and are easier to work with statistically. At the second level of analysis (group’s level), functional connectivity measures were calculated and compared using group-level statistical analysis, T-tests, and/or F-tests where appropriate, identifying and comparing the rsfMRI networks connected to each of the cerebellar regions. The standard of statistics for the results is displayed using a corrected false discovery rate (FDR) (*p* < 0.05) (multivariate statistics parametric (MVPA) omnibus test) [18]. With this approach, every connection is effectively subjected to a multivariate parametric general linear model analysis. Moreover, to better understand the functional connectivity within the brain, visualizations were generated using a matrix-based functional display. This approach, as defined in the CONN software, allows for the comprehensive analysis and interpretation of the complex relationships between different brain regions, ultimately providing valuable insights into their interactions and functional integration.

## 3. Results

**Figure 1:**
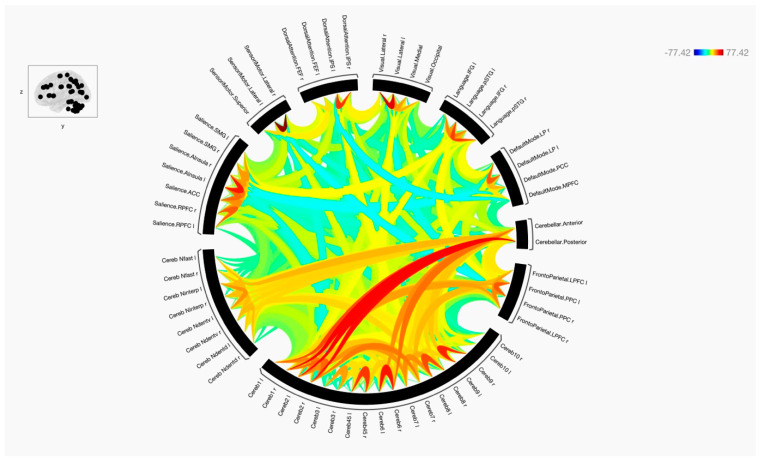
The functional connectivity of the selected seeds and target regions is shown at the group level. The lines of the connections in red indicate positive connectivity, while blue indicates negative connectivity. The colors of the lines are proportional to statistical strength, and the T-bar is shown in the top right-hand corner.

**Figure 2:**
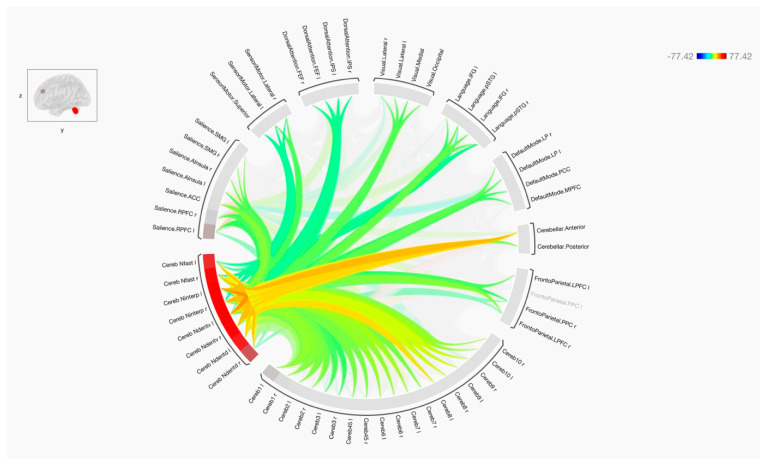
The functional connectivity of the cerebellar nuclei and target networks is shown at the group level. The lines of the connections in red indicate positive connectivity, while blue indicates negative connectivity. The colors of the lines are proportional to statistical strength, and the T-bar is shown in the top right-hand corner.

**Figure 3:**
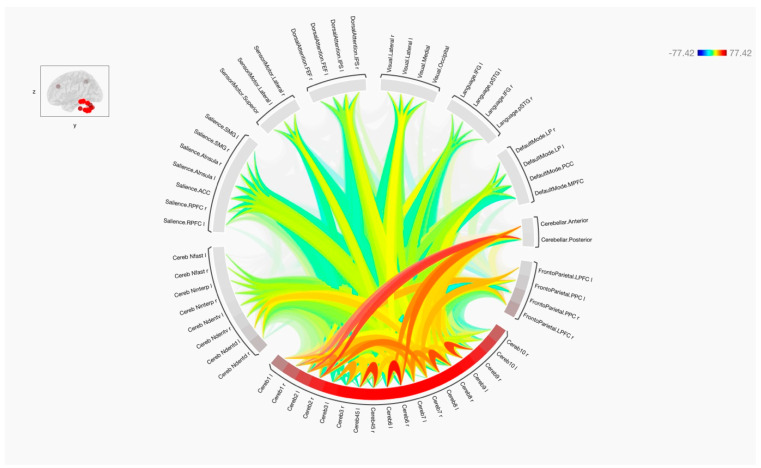
The functional connectivity of the cerebellar lobules and target regions is shown at the group level. The lines of the connections in red indicate positive connectivity, while blue indicates negative connectivity. The colors of the lines are proportional to statistical strength, and the T-bar is shown in the top right-hand corner.

Overall, the results of the functional connectivity are shown in Figure 1, Figure 2 and Figure 3. In general, there were strong connections among and between the cerebellar regions in both hemispheres. It is noticed that lobules of the anterior cerebellum have strong connections with each other, while lobules of the posterior cerebellum have strong connections with each other. One of the interesting results was the lack of functional connection seen with cerebellar lobules 3. Additionally, cerebellar nuclei have strong connections with each other in both hemispheres. It is also noted that the posterior cerebellum, especially lobules 8–10, has an increase in functional integrations with all the cerebellar nuclei.

**Figure 4:**
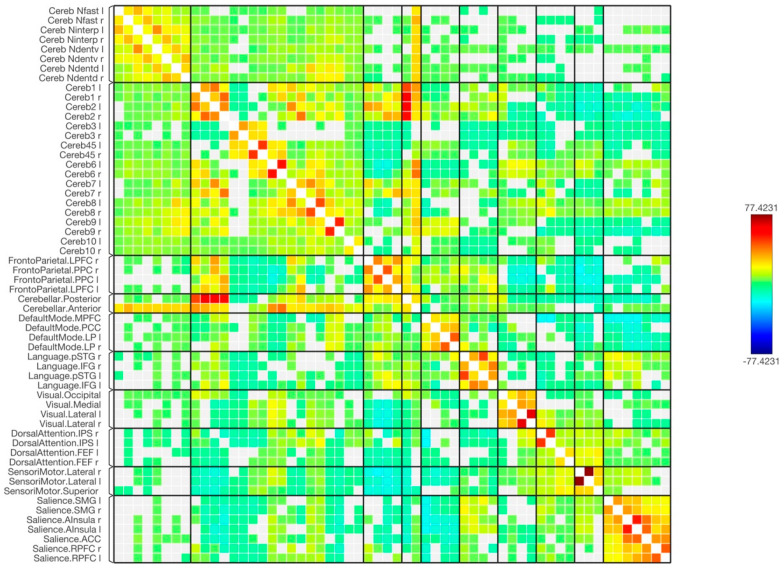
The matrix of the functional connectivity of the cerebellar lobules and their nuclei as well as the targeted brain networks is shown along with the statistical indications of the strength of the connection.

**Figure 5:**
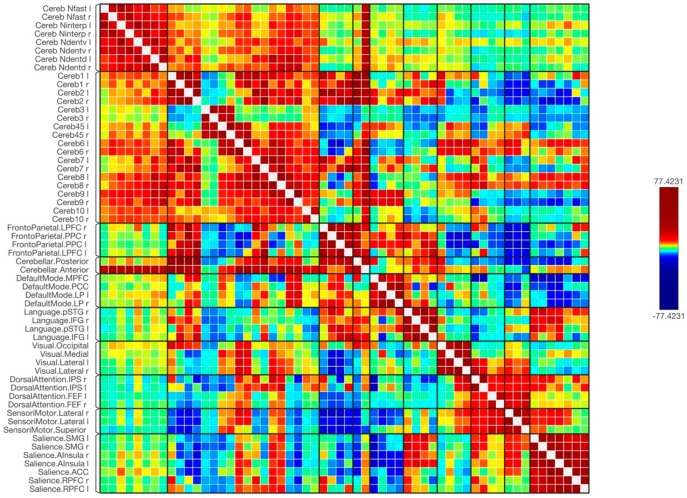
The matrix of the functional connectivity of the cerebellar lobules and their nuclei as well as the targeted brain networks is shown along with the statistical indications of the strength of the connection. In this figure, the connectivity was set with a statistical equalizer threshold (same threshold with a fixed color bar) to easily distinguish strong, weak, and negative connections.

Figure 4 and Figure 5 show the results of functional connectivity between the ten cerebellar lobules and four nuclei with major brain networks as a matrix of statistical connections between the selected brain regions. When observing the functional connectivity between the cerebellar areas and different brain networks, the cerebellar lobules that were connected strongly with the sensorimotor network were lobules 4, 5, 6, and 8, as well as the ventral part of the dentate nuclei. The default mode network was positively connected to lobules 2, 4, 5, 6, 7, 9, and partially with lobule 10. The dentate nuclei were also connected with the default mode network, especially the dorsal part. A notable finding of this study was the strong positive connection between the frontoparietal network and lobules 1, 2, and 7. Only the dorsal part of the deep cerebellar dentate nuclei was connected to this network. Looking at the integrations between the cerebellar lobules and nuclei with the language network, almost the same observation is seen as with the frontoparietal network. Here, lobules 1 and 2, parts of lobules 6 and 7, as well as left ventral and dorsal dentate nuclei were strongly connected to the language network. The visual network, including all its regions, was connected strongly to lobules 1, 6, and 8 bilaterally, and to the left hemisphere of lobule 9. This network was also connected to the left ventral dentate nucleus and right dorsal dentate nucleus. Parts of the visual network were also connected to lobules 4, 5, and 10, as well as the Interposed (emboliform and globose) and fastigial nuclei. Additionally, the dorsal attention network and its respective regions were strongly connected to lobules 6 bilaterally. This network was also partially connected to lobules 4, 5, 7, 8, and 10. The strong connection was obvious with the intraparietal sulcus. Moreover, the salience network was strongly connected to several cerebellar regions, including lobules 6 and 8, as well as the left Interposed (emboliform and globose) and right ventral dentate nuclei. Furthermore, negative correlations (anti-correlations) were observed among different cerebellar regions with several functional brain networks, including the frontoparietal, language, dorsal attention, and salience networks. These negative correlations were observed more frequently when using cerebellar lobules 3 and 9 as seeds and were less evident within the cerebellar nuclei.

## 4. Discussion

This study used a large cohort to investigate the functional connectivity between cerebellar lobules and deep cerebellar nuclei with eight regional network functions using rsfMRI with functional integration. The aim of this study was to investigate the similarities and differences between these cerebellar lobules and nuclei with functionally related resting state networks in a single, large cohort study and to determine whether the cerebellum is only involved in mediating and controlling motor functions or whether it has more complex functional involvement in other higher-order functions, as reported in other recent task fMRI studies [17,25]. By examining this extensive dataset, the researchers sought to provide a comprehensive understanding of the cerebellum’s diverse roles, further elucidating its contributions to cognitive, emotional, and sensory processing in addition to its well-known motor functions. This investigation aimed to clarify the cerebellum’s involvement in various networks and potentially expand our knowledge of its functional significance in the human brain.

The results of this study indicated that these cerebellar regions were linked to several functional networks in the brain in similar ways; however, there were differences in how they were linked to these networks and in the existence of functional connections between them. This study demonstrated the significant functional roles and involvement of the anterior and posterior cerebellum. The results are consistent with earlier research that reported the significance of these cerebellar regions in influencing a number of brain functions [16]. The findings also showed how the four cerebellar nuclei have significant connections with several cerebellar lobules, indicating the important functional roles as well of these nuclei [12,13,14,26]. There were increased functional integrations, especially within the dentate nuclei with most of the functional brain networks, which indicates the significant roles of these deep nuclei in a variety of different functions, including motor and non-motor.

In addition, the findings reveal that cerebellar lobules 4 and 5 are more related to sensorimotor networks and the default-mode networks. Indeed, the involvement and connections between the anterior cerebellum and the different sensorimotor areas are not surprising as this has been demonstrated using several task-related fMRI experiments, and the topology of the cerebellum is well known [5,15]. The unexpected result of this study was the increased involvement of these lobules in the default-mode network. The involvement of the default-mode network was also seen with several cerebellar nuclei, including the ventral and dorsal dentate nuclei. The medial prefrontal cortex, lateral parietal cortex, and posterior cingulate cortex are three interconnected brain areas that make up the default-mode network. These interconnected brain areas typically exhibit distinctive patterns compared with other networks [19]. The default-mode network is typically notably altered in neurological illnesses and is associated with intrinsic changes [20,21,22]. In addition, studies have shown that this network may be involved in motor-related functions, including motor imagery and learning [23]. These results highlight the significance of the cerebellar connections to the default mode network and suggest a potential role for the default mode network in cerebellum-controlled brain activities.

An additional observation in this study was related to cerebellar lobules 6 and 8, which were not only strongly connected to sensorimotor regional networks but were also integrated with higher-order complex networks, such as salience, dorsal attention, and visual networks. To further elucidate these findings, different task-related fMRI studies could be applied to investigate how the cerebellum behaves during these tasks. For example, previous studies have shown that lobule 6 is not only engaged during simple motor tasks, but it tends to be engaged during complex motor tasks, such as tool usage, squeezing balls with different force levels, and motor planning [27,28]. Furthermore, lobule 6 has been shown to be involved in higher-order, cognitive, and non-motor functions, including reading, working memory, and executive functions [16]. These findings could explain the greater functional integrations of bilateral lobules 6 and 8 of the cerebellum. This is especially the case in the attention and salience networks, where it has been demonstrated that the salience network is connected to a variety of sophisticated brain functions, including communication, social behavior, and self-awareness [29,30].

One of the interesting observations seen in this study is the strong involvement of the deep cerebellar nuclei with several functional-related brain networks. This was especially the case when looking at functional integrations between the dentate nuclei and the default mode, language, visual and salience networks. This observation highlights the importance of the deep cerebellar nuclei, including the dentate nucleus, in cognitive and affective processes beyond motor control. The strong involvement of the dentate nucleus in functional networks beyond the motor system may suggest that it plays a crucial role in the coordination of complex cognitive and affective processes [13,16,17,28,29].

The functional integration between the dentate nucleus and the default mode network is particularly noteworthy. The default mode network is a network of brain regions that is most active during passive or self-referential tasks, such as mind-wandering, daydreaming, and introspection [19,20,30]. The dentate nucleus has been shown to be involved in error detection and correction, which are important for maintaining accuracy in complex motor tasks [31,32,33]. The functional integration between the dentate nucleus and the default mode network could suggest that these two regions work together to monitor internal states, thoughts, and emotions and adjust behavior accordingly.

Similarly, the involvement of the dentate nucleus in the language network suggests a role in language processing, perhaps contributing to the formation and maintenance of internal representations of language. The dentate nucleus has also been shown to be functionally connected to the visual network, suggesting a role in visual processing, such as object recognition, spatial awareness, and attention [9,18,28]. Additionally, the dentate nucleus’s involvement in the salience network is consistent with its role in monitoring internal and external states and switching between different task demands. The salience network is thought to play a crucial role in attention allocation by detecting relevant and important stimuli and directing attention accordingly [26,27,34].

One limitation of this study is the use of a relatively larger voxel size compared to the small size of the deep cerebellar nuclei, as well as smoothing with an 8 mm kernel. However, recent studies have suggested that when investigating regional-to-regional resting functional integration analysis, there are fewer differences between larger or smaller smoothing kernel voxels [31]. One of the most important considerations that should be taken into account in future studies is the use of a probabilistic dedicated cerebellar atlas to accurately define detailed cerebellar regions based on their cytoarchitectonic properties [24,32,33,34]. In addition, the limitation in our sample, where 72% of the participants are women is acknowledged. Although our findings may be less affected by the gender imbalance based on the available literature, we emphasize the need for further research with more balanced samples to better understand the potential impact of gender on cerebellar functional connectivity. Furthermore, in this study, several negative functional connectivities were observed between the cerebellar regions with other brain networks. Negative correlation, in the context of functional connectivity, refers to an inverse relationship between the activity of two brain regions. When one region’s activity increases, the other region’s activity decreases, and vice versa. This may reflect competitive or inhibitory interactions between brain regions, as they can serve distinct or sometimes opposing functions. Interpreting negative correlations in functional connectivity studies can be challenging for several reasons, including understanding the physiological basis, whether they are context dependency, related to Inter-individual variability, or related to some sort of artifacts [35,36,37,38]. As our understanding of the neurobiological mechanisms underlying negative functional connectivity continues to evolve, we may be better equipped to interpret these findings in future studies. Future studies investigating the functional connectivity of the cerebellum should consider adopting voxel-based analysis methods to provide a more detailed and accurate understanding of functional boundaries and connectivity patterns. Examining the cerebellum at a voxel resolution may help identify subtle variations in functional connections and activity patterns that are not discernible at the lobular level. Furthermore, the implementation of advanced neuroimaging techniques and machine learning algorithms for the analysis of voxel-level data could reveal novel insights into the cerebellum’s involvement in cognitive processing, sensorimotor integration, and other brain functions, ultimately contributing to a more comprehensive understanding of this complex brain region.

## 5. Conclusions

The functional connections between several cerebellar lobules and the main networks of the brain were compared in this study. The results demonstrated that cerebellar lobules have heterogeneous functional integrations with various functional networks despite having significant functional connectivity between them. The anterior cerebellum (lobules 4 and 5) is more connected to sensorimotor-related networks, while the posterior cerebellum (lobule 6) is connected to the motor and higher-order, complex functional nonmotor networks. In addition, this study revealed the importance of anatomical identification of cerebellar areas, and future studies should consider identifying all lobules constituting the anterior and posterior cerebellum. Furthermore, this study highlighted the strong involvement of the deep cerebellar nuclei, particularly the dentate nucleus, in various functional networks, emphasizing their crucial role in cognitive and affective processes beyond motor control. These findings underscore the importance of investigating both cerebellar lobules and nuclei to gain a comprehensive understanding of the cerebellum’s diverse roles in human brain function.

## Data Availability

The data sample were taken from the Cambridge-Buckner data sample (open access) (http://fcon_1000.projects.nitrc.org (accessed on 9 April 2022)).

## Abbreviations

1. Default Mode Network (DMN):	
MPFC:	Medial Prefrontal Cortex
LP (L):	Left Lateral Parietal Cortex
LP (R):	Right Lateral Parietal Cortex
PCC: Posterior Cingulate Cortex	
2. SensoriMotor Network (SMN):	
Lateral (L):	Left Lateral Sensorimotor Cortex
Lateral (R):	Right Lateral Sensorimotor Cortex
Superior:	Superior Sensorimotor Cortex
3. Visual Network (VN):	
Medial:	Medial Visual Cortex
Occipital:	Occipital Visual Cortex
Lateral (L):	Left Lateral Visual Cortex
Lateral (R):	Right Lateral Visual Cortex
4. Salience Network (SN):	
ACC:	Anterior Cingulate Cortex
AInsula (L):	Left Anterior Insula
AInsula (R):	Right Anterior Insula
RPFC (L):	Left Rostral Prefrontal Cortex
RPFC (R):	Right Rostral Prefrontal Cortex
SMG (L):	Left Supramarginal Gyrus
SMG (R):	Right Supramarginal Gyrus
5. Dorsal Attention Network (DAN):	
FEF (L):	Left Frontal Eye Field
FEF (R):	Right Frontal Eye Field
IPS (L):	Left Intraparietal Sulcus
IPS (R):	Right Intraparietal Sulcus
6. FrontoParietal Network (FPN):	
LPFC (L):	Left Lateral Prefrontal Cortex
PPC (L):	Left Posterior Parietal Cortex
LPFC (R):	Right Lateral Prefrontal Cortex
PPC (R):	Right Posterior Parietal Cortex
7. Language Network (LN):	
IFG (L):	Left Inferior Frontal Gyrus
IFG (R):	Right Inferior Frontal Gyrus
pSTG (L):	Left Posterior Superior Temporal Gyrus
pSTG (R):	Right Posterior Superior Temporal Gyrus
8. Cerebellar Network (CN):	
Anterior:	Anterior Cerebellum
Posterior:	Posterior Cerebellum
Ndentv:	Dentate Nucleus Ventral
Ndentd:	Dentate Nucleus Dorsal
Ninterp: Interposed Nucleus, which can be further divided into:	
a. Globose Nucleus (GN)	
b. Emboliform Nucleus (EN)	
Nfast:	Fastigial Nucleus

## References

[1] Stoodley C.J., Schmahmann J.D. (2009). Functional Topography in the Human Cerebellum: A Meta-Analysis of Neuroimaging Studies. Neuroimage.

[2] Bernard J., Seidler R.D., Hassevoort K.M., Benson B.L., Welsh R.C., Wiggins J.L., Jaeggi S.M., Buschkuehl M., Monk C.S., Jonides J. (2012). Resting State Cortico-Cerebellar Functional Connectivity Networks: A Comparison of Anatomical and Self-Organizing Map Approaches. Front. Neuroanat..

[3] Stoodley C.J., Valera E.M., Schmahmann J.D. (2010). An Fmri Study of Intra-Individual Functional Topography in the Human Cerebellum. Behav. Neurol..

[4] Stoodley C.J. (2012). The cerebellum and Cognition: Evidence from Functional Imaging Studies. Cerebellum.

[5] Stoodley C.J., Valera E.M., Schmahmann J.D. (2012). Functional Topography of the Cerebellum for Motor and Cognitive Tasks: An Fmri Study. NeuroImage.

[6] King M., Rauch H.G., Stein D.J., Brooks S.J. (2014). The Handyman’s Brain: A Neuroimaging Meta-Analysis Describing the Similarities and Differences Between Grip Type and Pattern in Humans. Neuroimage.

[7] Tomlinson S.P., Davis N.J., Morgan H.M., Bracewell R.M. (2014). Cerebellar Contributions to Verbal Working Memory. Cerebellum.

[8] Biswal B., Zerrin Yetkin F., Haughton V.M., Hyde J.S. (1995). Functional Connectivity in the Motor Cortex of Resting Human Brain Using Echo-Planar Mri. Magn. Reson. Med..

[9] Biswal B.B., Mennes M., Zuo X.N., Gohel S., Kelly C., Smith S.M., Beckmann C.F., Adelstein J.S., Buckner R.L., Colcombe S. (2010). Toward Discovery Science of Human Brain Function. Proc. Natl. Acad. Sci. USA.

[10] Ito M. (2008). Control of Mental Activities by Internal Models in the Cerebellum. Nat. Rev. Neurosci..

[11] Schlerf J., Wiestler T., Verstynen T., Diedrichsen J. (2014). Big Challenges from the Little Brain—Imaging the Cerebellum. Adv. Brain Neuroimaging Top. Health Dis. Methods Appl..

[12] Diedrichsen J., Maderwald S., Küper M., Thürling M., Rabe K., Gizewski E.R., Ladd M.E., Timmann D. (2011). Imaging the Deep Cerebellar Nuclei: A Probabilistic Atlas and Normalization Procedure. NeuroImage.

[13] Küper M., Dimitrova A., Thürling M., Maderwald S., Roths J., Elles H.G., Gizewski E.R., Ladd M.E., Diedrichsen J., Timmann D. (2011). Evidence for a Motor and a Non-Motor Domain in the Human Dentate Nucleus—An Fmri Study. NeuroImage.

[14] Küper M., Wünnemann M.J., Thürling M., Stefanescu R.M., Maderwald S., Elles H.G., Göricke S., Ladd M.E., Timmann D. (2014). Activation of the Cerebellar Cortex and the Dentate Nucleus in a Prism Adaptation Fmri Study. Hum. Brain Mapp..

[15] Alahmadi A., Pardini M., Samson R., Friston K., Toosy A., D’Angelo E., Wheeler-Kingshott C. The Healthy Human Cerebellum Engaging in Complex Patterns: An Fmri Study. Proceedings of the International Society for Magnetic Resonance in Medicine.

[16] Stoodley C.J., Schmahmann J.D. (2018). Functional Topography of the Human Cerebellum. Handb. Clin. Neurol..

[17] Habas C. (2021). Functional Connectivity of the Cognitive Cerebellum. Front. Syst. Neurosci..

[18] Whitfield-Gabrieli S., Nieto-Castanon A. (2012). Conn: A Functional Connectivity Toolbox for Correlated and Anticorrelated Brain Networks. Brain Connect..

[19] Friston K.J., Holmes A.P., Worsley K.J., Poline J.P., Frith C.D., Frackowiak R.S. (1995). Statistical Parametric Maps in Functional Imaging: A General Linear Approach. Hum. Brain Mapp..

[20] Calhoun V.D., Wager T.D., Krishnan A., Rosch K.S., Seymour K.E., Nebel M.B., Mostofsky S.H., Nyalakanai P., Kiehl K. (2017). The Impact of T1 Versus EPI Spatial Normalization Templates for Fmri Data Analyses.

[21] Ashburner J., Friston K. (1997). Multimodal Image Coregistration and Partitioning—A Unified Framework. Neuroimage.

[22] Ashburner J., Friston K.J. (2005). Unified Segmentation. Neuroimage.

[23] Tellmann S., Bludau S., Eickhoff S., Mohlberg H., Minnerop M., Amunts K. (2015). Cytoarchitectonic Mapping of the Human Brain Cerebellar Nuclei in Stereotaxic Space and Delineation of Their Co-Activation Patterns. Front. Neuroanat..

[24] Eickhoff S.B., Stephan K.E., Mohlberg H., Grefkes C., Fink G.R., Amunts K., Zilles K. (2005). A New SPM Toolbox for Combining Probabilistic Cytoarchitectonic Maps and Functional Imaging Data. Neuroimage.

[25] Guell X., Schmahmann J.D., Gabrieli J.D., Ghosh S.S. (2018). Functional Gradients of the Cerebellum. Elife.

[26] Habas C. (2010). Functional Imaging of the Deep Cerebellar Nuclei: A Review. Cerebellum.

[27] Schlerf J.E., Verstynen T.D., Ivry R.B., Spencer R.M. (2010). Evidence of a Novel Somatopic Map in the Human Neocerebellum during Complex Actions. J. Neurophysiol..

[28] Imamizu H., Kawato M. (2009). Brain Mechanisms for Predictive Control by Switching Internal Models: Implications for Higher-Order Cognitive Functions. Psychol. Res. PRPF.

[29] Uddin L.Q. (2016). Salience Network of the Human Brain.

[30] Seeley W.W. (2019). The Salience Network: A Neural System for Perceiving and Responding to Homeostatic Demands. J. Neurosci..

[31] Alahmadi A.A. (2021). Effects of Different Smoothing on Global and Regional Resting Functional Connectivity. Neuroradiology.

[32] Diedrichsen J. (2006). A Spatially Unbiased Atlas Template of the Human Cerebellum. NeuroImage.

[33] Diedrichsen J., Balsters J.H., Flavell J., Cussans E., Ramnani N. (2009). A Probabilistic MR Atlas of the Human Cerebellum. NeuroImage.

[34] Amunts K., Kedo O., Kindler M., Pieperhoff P., Mohlberg H., Shah N.J., Habel U., Schneider F., Zilles K. (2005). Cytoarchitectonic Mapping of the Human Amygdala, Hippocampal Region and Entorhinal Cortex: Intersubject Variability and Probability Maps. Anat. Embryol..

[35] Passamonti L., Cerasa A., Liguori M., Gioia M.C., Valentino P., Nistico R., Quattrone A., Fera F. (2009). Neurobiological Mechanisms Underlying Emotional Processing in Relapsing-Remitting Multiple Sclerosis. Brain J. Neurol..

[36] Murphy K., Fox M.D. (2017). Towards a Consensus Regarding Global Signal Regression for Resting State Functional Connectivity MRI. Neuroimage.

[37] Chen G., Chen G., Xie C., Li S.J. (2011). Negative Functional Connectivity and Its Dependence on the Shortest Path Length of Positive Network in the Resting-State Human Brain. Brain Connect..

[38] Shibasaki H. (2012). Cortical Activities Associated with Voluntary Movements and Involuntary Movements. Clin. Neurophysiol. Off. J. Int. Fed. Clin. Neurophysiol..

